# A Reversal in Fortunes: A Case of Triumphant Retrograde Intubation

**DOI:** 10.7759/cureus.100274

**Published:** 2025-12-28

**Authors:** Vishal Tharani, Nickolas Meier

**Affiliations:** 1 Emergency Medicine, OhioHealth Doctors Hospital, Columbus, USA

**Keywords:** airway intubation, difficult airway management, emergency medicine resuscitation, in hospital cardiac arrest, rapid sequence intubation (rsi), retrograde intubation, surgical cricothyrotomy

## Abstract

A 48-year-old male presented to the emergency department in respiratory distress, with a history of poorly controlled asthma and a remote history of pulmonary embolus. After failing a non-invasive ventilation trial and numerous treatments for presumed status asthmaticus, he was planned for intubation. During the procedure, a large supraglottic mass was visualized, obstructing the airway and preventing successful orotracheal intubation via video laryngoscopy. Securing the airway was complicated by cardiac arrest, likely hypoxia-driven. The patient underwent a successful retrograde intubation followed by post-cardiac arrest stabilization. This case highlights the technique and its role in difficult airway management.

## Introduction

Oral endotracheal intubation, performed via direct visual or video-assisted laryngoscopy, is the standard benchmark for airway management. When this approach becomes unobtainable, is contraindicated, or fails, and the patient reaches a CICO (“can’t intubate and can’t oxygenate”) scenario, a different maneuver for airway access needs to be considered [1]. Cricothyrotomy and retrograde intubation are two alternative techniques in the difficult airway toolbox that clinicians should be familiar with when managing critically ill patients.

Retrograde intubation involves using a central venous J-shaped catheter guidewire, which is fed into an 18-gauge needle inserted through the cricothyroid membrane and directed cephalad until the wire spontaneously exits the mouth or nose. This lead then serves as a guide to slide an endotracheal tube over the wire and down the oral or nasal pharynx into the trachea [2]. This procedure was developed in 1960 by two otolaryngologists to aid in airway management during oral cancer surgery in a premeditated fashion. Since then, it has been employed in cases of complex airway management, including after multiple failed attempts by an experienced provider, situations of severe facial trauma, cervical spine injury, or distorted anatomy where direct visualization or other conventional intubation methods may be compromised [2,3].

Although retrograde intubation has a well-established role in managing difficult airways, it is rarely used compared to cricothyrotomy, which involves creating a surgical opening through the cricothyroid membrane followed by a gum-elastic bougie and subsequent endotracheal tube to establish a patent airway [4,5]. Compared to other rescue measures, such as surgical tracheostomy and cricothyrotomy, it is less invasive, and patients have reported minimal post-procedural discomfort from the procedure. Yet, unfamiliarity with the procedure has been a limiting step in becoming a more utilized procedure [2,6]. This case report discusses the successful use of retrograde intubation in an emergency department during an unanticipated difficult airway following an attempted cricothyrotomy, highlighting key steps, potential complications, and the circumstances that led to the choice of this approach. The report emphasizes the importance of maintaining proficiency in various airway management techniques to enhance patient outcomes in critical situations, particularly when first-line methods are ineffective or impractical.

## Case presentation

A 48-year-old male presented to the emergency department (ED) via emergency medical services (EMS) for 5 days of progressive shortness of breath. His medical history was significant for poorly controlled asthma and a remote pulmonary embolism, for which he was no longer on anticoagulation. Upon arrival, the patient was tachycardic, tachypneic, and hypoxemic (Table 1). He exhibited increased work of breathing and diffuse expiratory wheezes on pulmonary examination, raising concern for status asthmaticus.

**Table 1:** Initial vital signs

Vital signs on presentation	
Blood Pressure	167/93 mm/Hg
Heart Rate	123 beats per minute
Respiratory Rate	36 breaths per minute
Pulse Oximetry	53% on room air

Immediate treatment was started with a 0.5-2.5 mg/3 mL nebulizer solution of ipratropium-albuterol, 125 mg of intravenous (IV) methylprednisolone, 2 g of IV magnesium sulfate, 1 mg of intramuscular (IM) epinephrine, and bi-level positive airway pressure. Despite these measures, his respiratory distress worsened, causing obtundation and the decision to intubate. Rapid sequence intubation was attempted with 20 mg of etomidate for sedation and 100 mg of rocuronium for muscle paralysis. A hyper-angulated video S4 laryngoscope was selected to visualize the vocal cords in preparation for passing a 7.5 mm endotracheal tube (ETT). Unfortunately, the tube could not pass through the vocal cords after multiple attempts due to an obstructing mass at the base of the tongue that extended inferiorly and overlaid the vocal cords. An initial attempt to intubate past the mass resulted in esophageal intubation, with subsequent failure to oxygenate and ventilate the patient. The tube was downsized to a 6.0 mm ETT in preparation for subsequent attempts. Second and third attempts were complicated by bleeding from the vascular mass, further obstructing the view. At this point, the on-call anesthesiologist was overhead paged to the ED to assist with the difficult airway.

A bag-valve-mask (BVM) resuscitation technique was used to assist the patient’s ventilation during the transition to definitive airway management. Unfortunately, while setting up for a cricothyrotomy, the patient suffered a cardiac arrest from asystole, likely from prolonged hypoxia. Still, return of spontaneous circulation (ROSC) was achieved after one round of advanced cardiac life support (ACLS), including high-quality cardiopulmonary resuscitation (CPR), 1 mg of IV epinephrine, and 50 mEq of sodium bicarbonate. Cardiac monitoring revealed ventricular tachycardia with a blood pressure of 96/58 mmHg. Amiodarone (150 mg) was administered, which led to conversion to sinus tachycardia. Further cardiac monitoring continued to show a sinus rhythm with resolution of tachycardia.

While performing ACLS, the neck was prepped with chlorhexidine for surgical cricothyrotomy. A scalpel was used to make a vertical incision through the skin, fascia, and cricothyroid membrane, and a 15Fr bougie was inserted through the opening to guide the placement of a 6.0 mm ETT. However, the supraglottic mass prevented the bougie from moving caudally, so it was redirected for retrograde intubation. The bougie was retracted, then was passed through the cricothyroid membrane incision and directed cephalad. Once visualized, an anesthesiologist at the head, utilizing an S4 video laryngoscope and a 6.0 mm ETT, gently guided the tube downward over the bougie, past the mass, and into the lower airway. The bougie was then simultaneously retracted from the mouth as the ETT was guided into the trachea. Bilateral breath sounds and improved oxygen saturations confirmed proper tube placement. Repeat vital signs demonstrated the patient’s improved clinical stability (Table 2).

**Table 2:** Repeat vital signs FiO2: fraction of inspired oxygen

Improvement in vital signs	
Blood Pressure	125/57 mm/Hg
Heart Rate	74 beats per minute
Respiratory Rate	26 breaths per minute
Pulse Oximetry	97% on 30% FiO2

**Figure 1:**
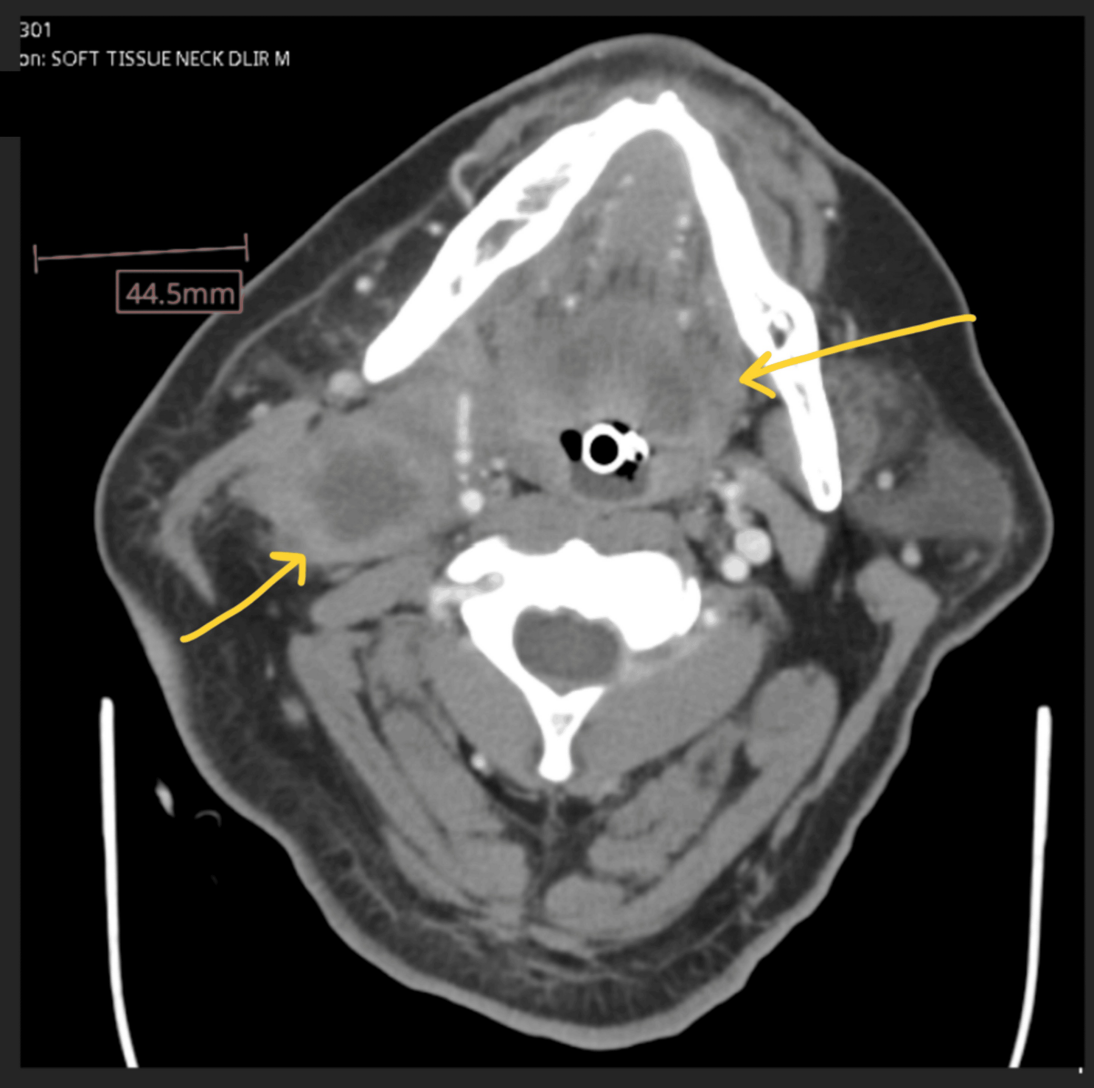
Axial view of CT soft tissue neck with contrast

**Figure 2:**
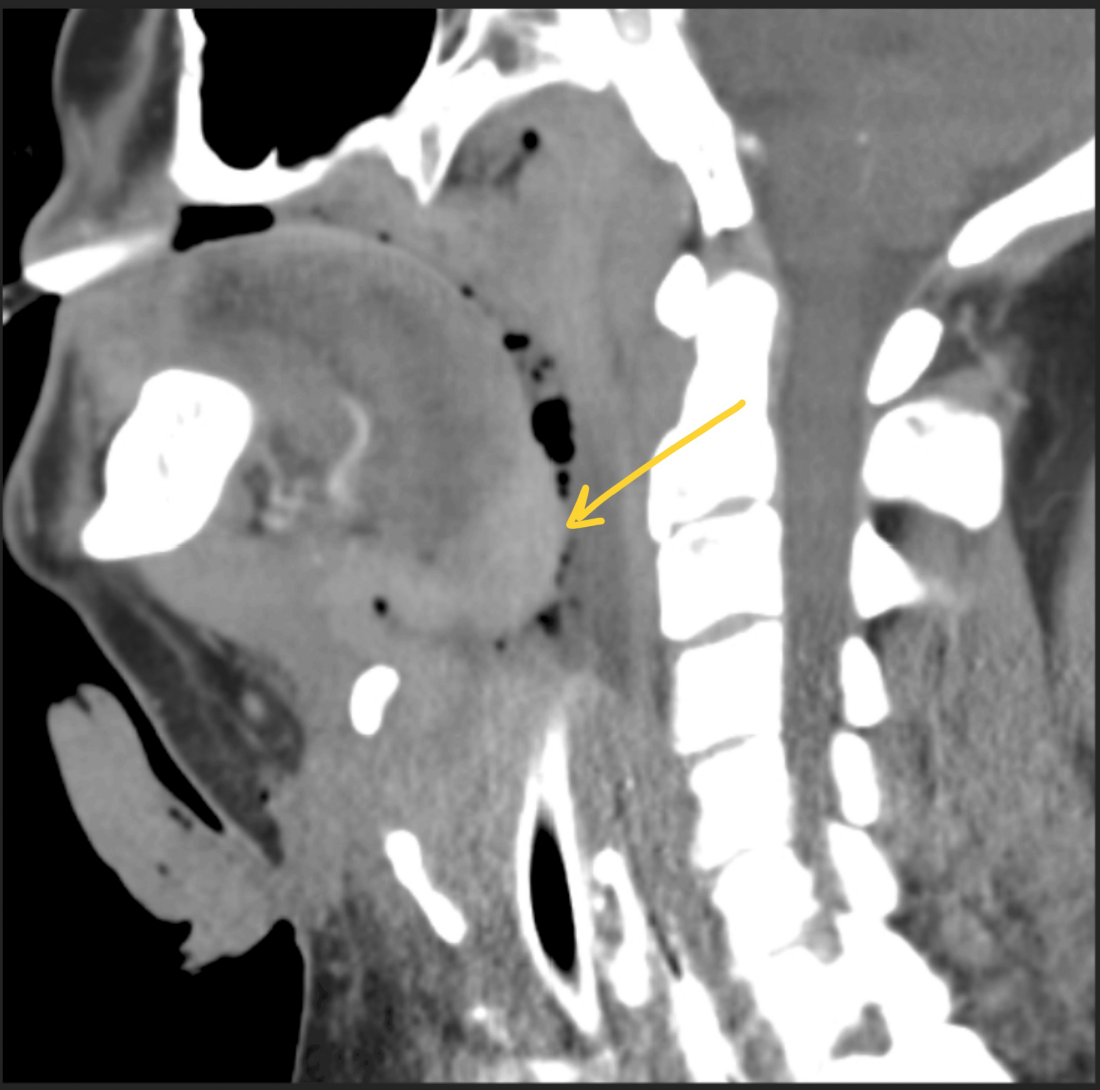
Sagittal view of CT soft tissue neck with contrast

Since the patient presented with tachycardia and tachypnea, and initial lab work showed leukocytosis with elevated lactic acid (Table 3), he received IV fluids following the sepsis protocol, along with broad-spectrum antibiotics (vancomycin and cefepime). Additionally, he required vasopressor support with norepinephrine (5 mcg/min) to maintain a mean arterial pressure (MAP) of greater than 65 mmHg. A ketamine infusion (0.5 mg/kg/hr) was started for sedation and for improved bronchodilation due to his concomitant status asthmaticus. Once stable, a contrast-enhanced computed tomography (CT) scan of the neck soft tissues was performed to examine the mass in detail. The CT revealed an enhancing, infiltrative mass at the base of the tongue, extending anteriorly and inferiorly into the larynx and involving the laryngeal and thyroid cartilages circumferentially. This raised suspicion for primary squamous cell carcinoma (Figures 1, 2).

**Table 3:** Initial lab work Hgb: hemoglobin; HCT: hematocrit; BUN: blood urea nitrogen

Lab Test	Lab Results	Normal Range
WBC	17.02 K/mcl	4.50 - 11.00 K/mcL
Hgb	13.9 g/dL	12-16 g/dL
HCT	43.9 %	36-46%
Platelets	210 K/mcl	150-400 K/mcL
Lactic Acid	2.3 mmol/L	0.6-2.0 mmol/L
BUN	31 mg/dL	8-25 mg/dL
Creatinine	1.27 mg/dL	0.4-1.1 mg/dL
Sodium	135 mmol/L	135-145 mmol/L
Potassium	4.3 mmol/L	3.5-5.1 mmol/L
Chloride	92 mmol/L	98-108 mmol/L
Bicarbonate	34 mmol/L	21-32 mmol/L
Anion Gap	13 mmol/L	10-20 mmol/L

The patient was transferred to a tertiary care center, where an otolaryngologist evaluated him and confirmed the diagnosis of squamous cell carcinoma of the glottis. A tracheostomy and percutaneous gastrostomy tube were placed; he was discharged home two weeks later with an outpatient plan for chemotherapy and radiation therapy.

## Discussion

In this case, a 48-year-old male presented with respiratory distress symptoms suggestive of status asthmaticus. After failed treatments, rapid sequence intubation was attempted and was not anticipated to be difficult. Once the obstructive mass was visualized and traditional orotracheal intubation was unsuccessful, backup measures were initiated. Between cricothyrotomy and retrograde intubation, a review of the literature has shown a preference for the former due to familiarity and a belief of decreased invasiveness [2,6]. Additionally, retrograde intubation was first established as a planned procedure in the 1960s with an awake patient under regional anesthesia [5]. It is less thought of as an emergency procedure; however, it only requires a few tools and has few contraindications and a high level of skill retention [7].

National airway management guidelines recommend cricothyroidotomy as a rescue technique in CICO scenarios [1,8]. In our patient’s case, cricothyroidotomy was initially attempted; however, obstructing anatomy likely caused the bougie to advance upward into the larynx, preventing a secure airway. Similar scenarios occurred in other case reports, which all described the successful placement of an ETT using a retrograde intubation in an emergent fashion status post failed fiber optic orotracheal intubation, video-assisted orotracheal intubation, and surgical tracheostomy [1,6,8]. Due to obstructing anatomy and CICO scenarios that couldn’t be bolstered by adequate bagging measures, securing the airway was necessary, and, ultimately, this technique was successfully utilized.

Although traditionally described as using an S-guide for initial placement [1,2], the same procedure was accomplished with a gum-elastic bougie in this report. Compared to the referenced literature, this patient was treated in the emergency department as opposed to the operating room or intensive care unit. Like anesthesiologists, emergency physicians must be familiar with a variety of techniques for airway securement, given the need for quick action during emergent scenarios. Retrograde intubation has been proven to be a life-saving procedure and should become a mainstay in the difficult airway algorithm in the minds of emergency and critical care physicians.

## Conclusions

This case underscores the vital role of retrograde intubation as a key component of the airway management toolkit for emergency physicians confronted with challenging airway scenarios. In patients with limited mouth opening, maxillofacial trauma, or distorted anatomy, as in this case report, reliance on direct laryngoscopy alone may delay definitive airway control and heighten the risk of hypoxia and subsequent complications. Mastery of retrograde intubation provides a rapid, minimally invasive alternative that can improve first-pass success rates and reduce peri-intubation complications. Incorporating this technique into emergency medicine training curricula and regular simulation reinforces clinician confidence and procedural competence. Ultimately, proficiency in retrograde intubation broadens procedural flexibility and enhances patient safety in the emergency department.
